# The Complete Mitochondrial Genome of the *Dioscorea opposita* Thunb. cv. Tiegun, a Traditional Medicinal and Edible Crop

**DOI:** 10.3390/biology15020133

**Published:** 2026-01-12

**Authors:** Dan Zhu, Feng Feng, Xiaoyong Shi, Mengqi Tian, Zhixiang Chen, Jiewei Zhang

**Affiliations:** 1 School of Environmental Engineering, Yellow River Conservancy Technical University, Kaifeng 475004, China; zhudan@yrcti.edu.cn (D.Z.); fengfeng@yrcti.edu.cn (F.F.); 2025851312@yrctu.edu.cn (X.S.); tianmengqi@yrcti.edu.cn (M.T.); 2 Beijing Key Laboratory of Agricultural Genetic Resources and Biotechnology, Beijing Key Laboratory of Crop Molecular Design and Intelligent Breeding, Beijing Academy of Agriculture and Forestry Sciences, Beijing 100097, China; zzc2000123@126.com

**Keywords:** *Dioscorea opposita*, mitochondrial genome, RNA editing, phylogenetic analysis, *nad4*

## Abstract

This study reports the first complete mitochondrial genome of the traditional medicinal and edible crop, *D. opposita* (493,268 bp, 45.67% GC). We annotated 39 unique protein-coding genes (PCGs), which included 24 core mitochondrial genes and 15 variable genes, as well as 19 tRNA genes and 3 rRNA genes, along with 245 SSRs and multiple repeat sequences. The longest palindromic repeat measured 260 bp, while the longest forward repeat was 24,068 bp. Furthermore, 723 RNA editing sites were discovered, all involving C-to-U edits, with the *nad4* having the highest number of edits (60 sites in total). Comparative genomic and phylogenomic analyses revealed Tiegun yam conserved gene content but structural variations compared to other monocots, underscoring the role of repetitive sequences and recombination in shaping mitochondrial architecture and facilitating cytonuclear co-adaptation. These findings establish a crucial genomic foundation for understanding mitochondrial regulation of growth and metabolic traits in this important species, with implications for future molecular breeding and functional studies of medicinal compound biosynthesis.

## Simple Summary

This study reports the first complete mitochondrial genome of *Dioscorea opposita*, which is one of the medicinal and edible homologous crops and is rich in various nutrients and functional compounds. The genome was assembled into four circular contigs totaling 493,268 bp, with a GC content of 45.67%. Interestingly, the 60 C-to-U editing sites in *D. opposita nad4* constitute highest count so far documented for any crop species, implying a potentially pivotal role for this gene in energy metabolism and environmental adaptation. These findings provide valuable insights into the phylogenetic and gene function research of *Dioscorea* plants.

## 1. Introduction

*Dioscorea opposita* Thunb., a perennial vine of the Dioscoreaceae family, is both medicinal and edible, rich in bioactive compounds like polysaccharides, flavonoids, and saponins [1,2]. It offers significant nutritional and health benefits. Predominantly cultivated in China’s Henan and Hebei regions [3,4], Henan is the main producer of the renowned *D. opposita* cv. Huai shan yao. Among its variants, *D. opposita* cv. *Tiegun* (Tiegun yam) is the most extensively cultivated [5].The Tiegun yam is noted for its numerous fibrous roots, slightly darker skin, and rust-red spots, attributed to anthocyanin accumulation. Its rhizomes are rich in proteins, dietary fiber, minerals, polysaccharides, and allantoin, which provide pharmacological benefits such as anti-hyperglycemic and immunomodulatory effects [6,7,8].

Mitochondria, essential organelles in eukaryotic cells, are often termed the “powerhouses of the cell” due to their role in synthesizing adenosine triphosphate (ATP) via oxidative phosphorylation, thus supplying the energy necessary for cellular functions [9]. Beyond their role in energy metabolism, mitochondria participate in vital physiological processes, including fatty acid metabolism, regulation of calcium ion homeostasis, and apoptosis [10,11]. Recent research indicates that plant mitochondria not only facilitate energy metabolism but also function as a “command center” influencing growth, development, and stress responses. A notable instance of mitochondrial genomic influence on phenotypic traits is cytoplasmic male sterility (CMS) [12]. This condition generally results from mitochondrial genomic recombination, producing chimeric genes that encode specific toxic proteins. In rice, for example, the *WA352* gene encodes a protein that targets mitochondria in tapetal cells, disrupting their normal function. This disruption leads to the accumulation of reactive oxygen species (ROS) and triggers programmed cell death, culminating in pollen abortion and male sterility [13,14,15,16,17,18]. The alternative oxidase (AOX) pathway is another key mitochondrial mechanism in stress adaptation [19]. When the primary respiratory chain is compromised, AOX acts as an “electron sink” redirecting electrons from the cytochrome pathway. Although this does not produce ATP, it scavenges excess reducing equivalents, mitigates oxidative damage, and rebalances carbon and energy metabolism. This enhances plant survival and recovery under adverse conditions such as drought and high temperatures. For instance, *A. thaliana* mutants lacking the *AOX1A* gene show increased sensitivity to drought stress, highlighting this pathway’s role in stress resistance [19].

The mitochondrial genome of Tiegun yam has not been characterized, despite the well-established roles of mitochondria in energy metabolism and stress adaptation. This knowledge gap impedes understanding in several critical areas. Firstly, the regulatory impact of mitochondrial genomic features on the biosynthesis of pharmacologically active compounds like polysaccharides and saponins in this medicinal plant is unknown. Secondly, elucidating the molecular mechanisms behind its environmental stress adaptation requires knowledge of its mitochondrial genetic makeup. Additionally, the evolutionary trends of the mitochondrial genome in the Dioscoreaceae family and their implications for structure-function relationships remain unexplored. The absence of this essential genomic data also hampers the development of molecular breeding strategies to enhance both medicinal quality and stress tolerance in this significant crop. Therefore, a thorough characterization of the Tiegun yam mitochondrial genome is crucial to address these knowledge gaps and promote advancements in both fundamental research and agricultural practices.

The mitochondrial genome of the Tiegun yam was sequenced and annotated de novo in this study. Mitochondrial genomes play a critical role in regulating plant growth, development, and stress responses, making their characterization crucial for understanding cytonuclear interactions and the molecular basis of the yam’s geo-authenticity. The findings also have practical implications, as they can facilitate marker-assisted breeding for improved yield and disease resistance in this economically and medicinally important species. The study presents a comprehensive analysis of the Tiegun yam mitochondrial genome, including its genomic structure, repetitive elements, RNA editing sites, and codon usage bias. Comparative analyses with other monocots were conducted, and a phylogenomic tree was reconstructed to assess the evolutionary relationships within the monocot clade. These results, combined with ongoing research on Chinese yam germplasm, establish a genomic foundation for future investigations into cytonuclear coordination in the Tiegun yam.

## 2. Materials and Methods

### 2.1. Plant Material and Mitochondrial Genome Assembly

‘Tiegun yam’ from Dioscorea species were collected from the research greenhouse at Beijing Academy of Agriculture and Forestry Sciences (BAAFS) located in Haidian District, Beijing City, China. The species were formally identified by Dr. Dan Zhu. Voucher specimen has been deposited in the Beijing Crop Germplasm Resources Infrastructure, a component of the BAAFS, with deposition numbers BJCC1101,040,001 (*D. opposita*). The mitochondrial genome was initially reconstructed from long-read sequencing data. The Flye software (v2.9.3) was employed with default settings to directly assemble the long-reads data, resulting in graphical assembly outputs in GFA format. Subsequently, all assembled contigs in fasta format were utilized to construct libraries using makeblastdb [20]. The BLASTN prog. ram was then applied, with the mitochondrial gene of a closely related species (*Trichopus zeylanicus*, OR830326.1) serving as the query sequence, employing the parameters “-evalue 1*e*-5-outfmt 6-max_hsps 10-word_size 7-task blastn-short” to identify contig fragments containing mitochondrial genomes. Visualization of the GFA files was performed using Bandage software (v0.8.1) [21], and mitochondrial contigs were filtered based on the BLASTN results to generate a mitochondrial genome outline. Subsequently, minimap 2 software (v2.26-r1175) was utilized to align the long-reads and short-reads data with the mitochondrial contigs [22]. The aligned mitochondrial reads were then filtered, extracted, and stored separately for subsequent hybrid assembly. The final assembly of the mitochondrial genome was achieved through a hybrid assembly approach, combining the aforementioned short-reads and long-reads sequencing data. The Unicycler software (v0.4.7) [23], with default parameters, was employed for hybrid assembly to ultimately obtain the mitochondrial genome. Visualization of the mitochondrial genome was conducted once more using Bandage software (v0.8.1).

### 2.2. Gene Annotation

The mitochondrial genome was annotated with the angiosperm mitochondrial genome annotation tool PMGA (http://www.1kmpg.cn/pmga/ accessed on 22 October 2025) [24], utilizing 319 mitochondrial genomes selected from the reference database. The tRNAs within the mitochondrial genome were identified using the tRNAscan-SE software (v.2.0.11) [25], while mitochondrial rRNA sequences were annotated through the utilization of BLASTN software (v2.13.0) [26]. Any errors in mitochondrial genome annotation were rectified through manual intervention using the Apollo software (v1.11.8) [27]. Visualization of the mitochondrial genome mapping was achieved using the OGDRAW software (v1.3.1) [28].

### 2.3. Codon Usage Bias

The protein-coding sequences were extracted with PhyloSuite software (v1.1.16) [29]. Subsequently, the protein-coding genes of the mitochondrial genome underwent analysis for codon preference, with the RSCU values being determined using Mega software (v7.0) [30].

### 2.4. Repeat Sequence

Repeat sequences were systematically identified using MISA for microsatellites [31]. Tandem Repeats Finder for tandem repeats [32], and REPuter for dispersed repeats [33]. Visualization was performed using the Circos package (v0.69.9) [34].

### 2.5. Mitochondrial Genome Structure

The strategy to resolve repetitive regions in the genomic assembly involved aligning long reads to repetitive sequences. This alignment assessed the continuity of these regions, particularly highly repetitive ones, by determining if they were fully spanned. Based on this evidence, the most likely genome structure of *D. opposita* was inferred by identifying the correct path through the assembly graph.

### 2.6. Sequence Migration Analysis

The chloroplast genome was assembled using the GetOrganelle software (v1.7.7.0) [35], annotated with CPGAVAS2 [36], and the annotation results were refined using CPGView (https://github.com/cliu6688/CPGView accessed on 22 October 2025) [37]. Homologous sequences were analyzed via BLASTN (v2.13.0) [26], and the findings were visualized employing the Circos package (v0.69.9) [34].

### 2.7. Phylogenetic Analysis

Phylogenetically related species were selected, and their mitochondrial genomes were obtained. Shared genes were extracted using PhyloSuite (v1.1.16) [29], followed by multiple sequence alignment with MAFFT (v7.505) [38,39]. A maximum likelihood phylogenetic tree was constructed using IQ-TREE (v1.6.12) [40] with parameters “--alrt 1000 -B 1000”, and the results were visualized using ITOL (v6) [41].

### 2.8. RNA Sequencing and RNA Editing Event

Total RNA was isolated with the RNAsimple Total RNA Extraction Kit (DP419; TIANGEN, Beijing, China) following the manufacturer’s instructions. First-strand cDNA was synthesized with reverse transcriptase and random hexamers, followed by second-strand synthesis, end repair, adaptor, and size selection with AMPure XP beads (Beckman Coulter, Brea, CA, USA). Libraries were sequenced on an Illumina NovaSeq 6000 instrument (Illumina, Inc., San Diego, CA, USA) to generate 150-bp paired-end reads.

RNA editing sites in protein-coding genes were predicted using two complementary approaches: computational prediction with Deepred-Mt (v1.0) [42] (probability threshold > 0.9) and empirical validation through transcriptome mapping using REDItools (v2.0) [43] where transcriptomic data were available.

### 2.9. Synteny Analysis

Conserved homologous sequences, referred to as syntenic blocks, were identified using BLASTN (v2.13.0) with the following parameters: -value 1*e*-5, -word size 9, -gapopen cost = 5, -gapextend cost = 2, -reward score = 2, and -penalty = −3. Only syntenic blocks exceeding 500 bp were selected for further analysis. Pairwise comparisons of multiple synteny plots were generated using MCscanX (v1.0.0) based on the BLASTN (v2.13.0) results to depict the conserved syntenic blocks [44].

## 3. Results

### 3.1. Genomic Features of the D. Thunb. cv. Tiegun Mitochondrial Genome

**Figure 1:**
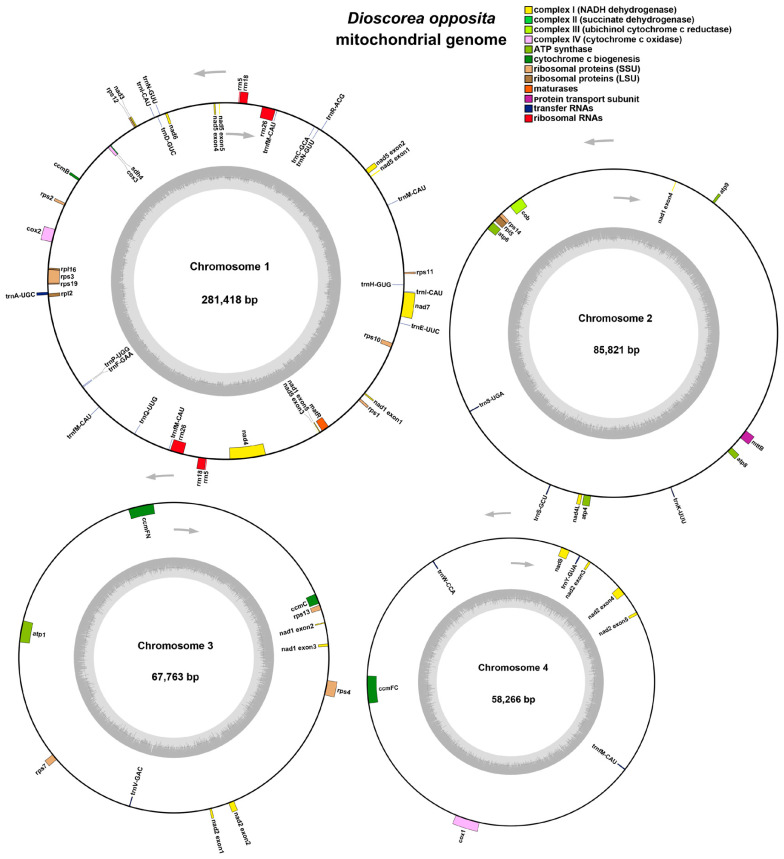
Circular representation of the Tiegun yam mitochondrial genomes. Genes transcribed clockwise are displayed on the outer circle, while those transcribed counterclockwise are on the inner circle. Genes belonging to different functional groups are color-coded.

The mitochondrial genome of the Tiegun yam exhibits a multi-branched conformation with an overall length of 493,268 bp and a GC content of 45.67% (Table 1). The mitochondrial genome of the *D. Thunb.* cv. Tiegun is characterized by a multi-branched structure, spanning 493,268 bp with a GC content of 45.67% (Table 1). Using long-read sequencing data and Bandage (v.8.1) visualization [21,45], the assembly was resolved into four distinct circular contigs (Figure S1). Genome annotation identified 39 unique protein-coding genes (Figure 1), comprising 24 core and 15 non-core genes, along with 19 tRNA genes (including 3 multicopy tRNAs) and 3 multicopy rRNA genes. The core gene set includes 5 ATP synthase genes (*atp1, atp4, atp6, atp8, atp9*), 9 NADH dehydrogenase genes (*nad1-nad7, nad9*), 4 cytochrome C biogenesis genes (*ccmB, ccmC, ccmFC, ccmFN*), 3 cytochrome C oxidase genes (*cox1-cox3*), 1 membrane transport protein gene (*mttB*), 1 maturase gene (*matR*), and 1 ubiquinol-cytochrome C reductase gene (*cob*). The non-core genes encompass 16 ribosomal protein genes and 1 succinate dehydrogenase gene (*sdh4*), with 3 from the large ribosomal subunit (*rpl2, rpl5, rpl16*) and 11 from the small ribosomal subunit (*rps1–rps4, rps7, rps10–rps14, rps19*) (Table 2).

### 3.2. Codon Usage Analysis of the PCGs

**Figure 2:**
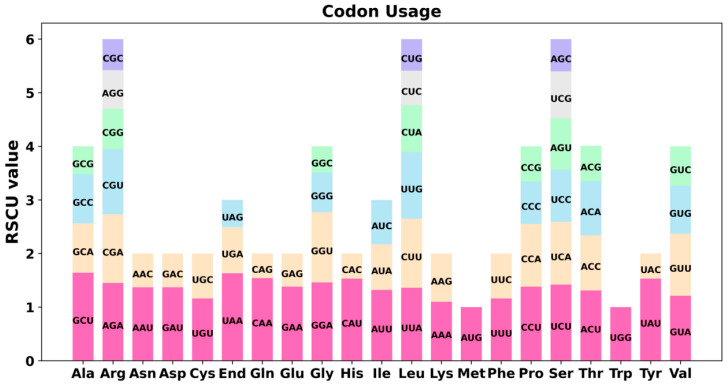
The Tiegun yam mitogenome relative synonymous codon usage (RSCU). The X-axis represents codon families, while RSCU values indicate the frequency of a specific codon relative to the expected frequency under uniform synonymous codon usage.

The eukaryotic genome contains 64 codons that code for 20 amino acids and three stop codons. Apart from methionine (Met) and tryptophan (Trp), most amino acids are represented by multiple codons. Codon usage bias varies significantly across different species and organisms, likely shaped by long-term evolutionary processes that have established a relatively stable intracellular environment. Therefore, in comparative genomic studies, codon bias is commonly assessed using the Relative Synonymous Codon Usage (RSCU) metric. In this study, we conducted a codon usage analysis of 39 protein-coding genes (PCGs) in the mitochondrial genomes of the Tiegun yam. The codon usage for each amino acid is presented in Table S1. RSCU values exceeding 1 are indicative of preferences for certain amino acids. Figure 2 illustrates the codon usage bias in mitochondrial PCGs, revealing a prevalent pattern except for the initiation codon AUG and the Trp codon (UGG), both having an RSCU value of 1. Notably, alanine (Ala) predominantly utilized the GCU codon with an RSCU value of 1.64. The termination codon UAA also exhibited a significantly high RSCU value. It is important to note that interpreting stop codons in terms of “usage preference” should be approached with caution since their primary function is termination rather than translation. Moreover, lysine (Lys) and phenylalanine (Phe) demonstrated maximum RSCU values below 1.2, indicating a lack of substantial codon usage bias based on the conventional criteria where RSCU values above 1.2 typically signify meaningful preference.

### 3.3. Repeat Sequence Analysis

**Figure 3:**
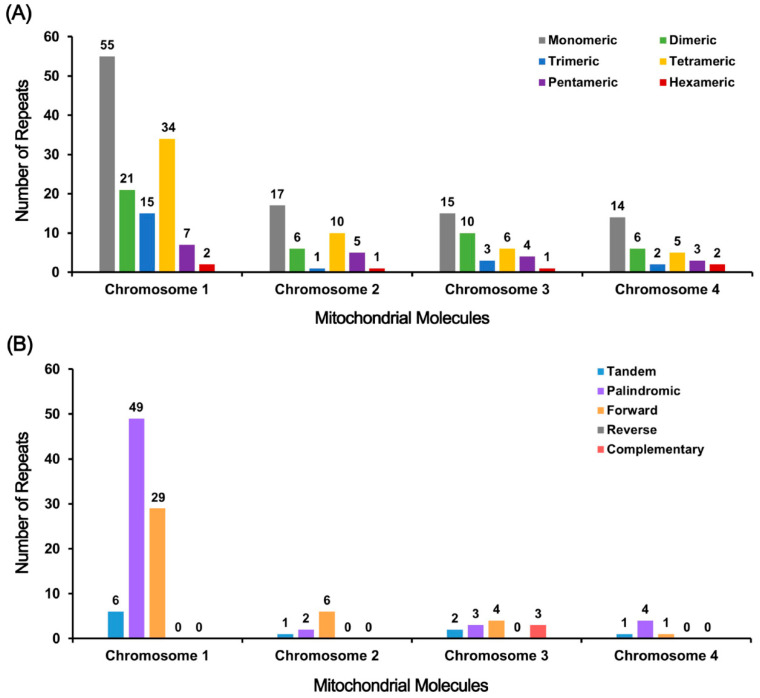
Detected repeats in the Tiegun yam. (**A**) The X-axis denotes mitochondrial molecules, while the Y-axis indicates the frequency of repetitive fragments. Monomeric SSRs are denoted by gray, dimeric SSRs by green, trimeric SSRs by blue, tetrameric SSRs by yellow, pentameric SSRs by purple, and hexameric SSRs by red. (**B**) the X-axis represents mitochondrial molecules, and the Y-axis represents the occurrence of repetitive fragments. Tandem repeats are shown in blue, palindromic repeats in purple, forward repeats in orange, reverse repeats in gray, and complementary repeats in red.

Eukaryotic and prokaryotic chromosomes contain repetitive DNA elements categorized into tandem repeats and dispersed repeats based on their spatial distribution [46]. An analysis of the Tiegun yam genome revealed significant amounts of both tandem and dispersed repeats on all four chromosomes (Tables S2–S13). Tandem repeats mainly consist of simple sequence repeats (SSRs) and satellite DNA, with a total of 245 SSRs identified. Monomeric and dimeric forms are prevalent, accounting for 56.72% on Chr1, 57.5% on Chr2, 64.10% on Chr3, and 62.50% on Chr4, indicating their widespread presence (Figure 3A). 10 tandem repeats were identified with high similarity levels (74–100%) and lengths ranging from 2 to 42 bp. 6 of these repeats were located on Chr1, showing the lowest similarity, while Chr2–4 displayed full matches but shorter lengths. A total of 101 dispersed repeat pairs were identified, primarily composed of palindromic and forward repeats, with reverse and complementary repeats being rare, with only 3 complementary pairs found on Chr3. Chr1 exhibited the highest number of dispersed repeats, including the longest forward repeat (24,775 bp) and palindromic repeat (276 bp), while the other chromosomes displayed fewer and shorter repeats (Figure 3B). These structural attributes may influence DNA stability, recombination events, and regulatory evolution, though their precise biological relevance requires further experimental validation. The remaining chromosomes featured relatively fewer and shorter dispersed repeats. In summary, the Tiegun yam genome displays a notable abundance of SSRs and dispersed repeats, particularly on Chr1, with tandem repeats being less prevalent but highly conserved. These structural characteristics are associated with the maintenance of genome stability, evolutionary adaptation, and functional regulation.

### 3.4. Homologous Fragments of Mitochondria and Chloroplasts

**Figure 4:**
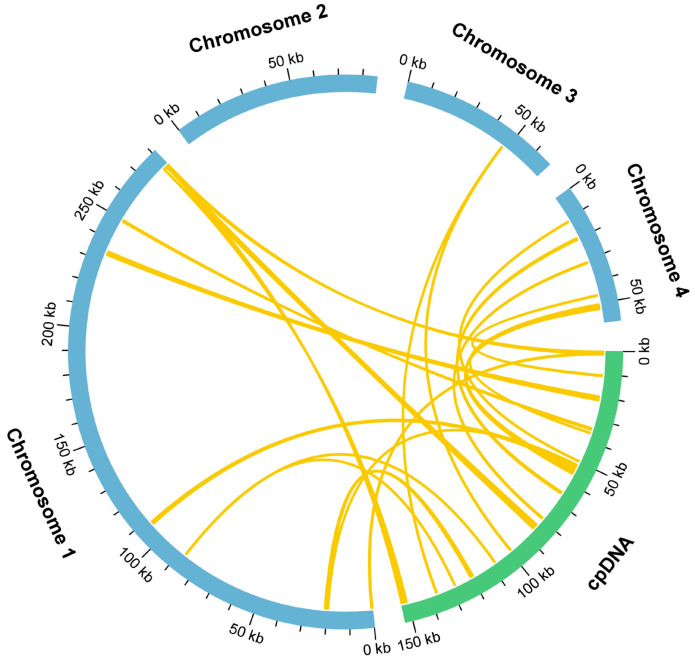
Schematic diagram of gene transfer between chloroplast and mitochondrial genomes in the Tiegun yam. The blue and green arcs represent the mitochondrial and chloroplast genomes, respectively. The yellow lines connecting the arcs indicate homologous genomic fragments.

During mitochondrial genome evolution, chloroplast-derived DNA fragments can undergo intracellular gene transfer events, leading to their integration. The length and sequence conservation of these fragments exhibit significant variation among species. In our study of the Tiegun yam, we detected 18 homologous fragments shared between the mitochondrial and chloroplast genomes through sequence similarity analysis (Figure 4, Table S14), totaling 10,086 bp. This accounts for 2.04% of the entire mitochondrial genome, a proportion consistent with the moderate level of plastid DNA integration observed in angiosperm mitochondrial genomes. This finding suggests the presence of conserved mechanisms of horizontal transfer despite lineage-specific evolutionary paths. Among these fragments, MTPT3 is the longest at 1,555 bp. Annotation of these regions identified eight intact genes, including two protein-coding genes (ndhA and rpl23) and 6 tRNA genes (trnfM-CAU, trnH-GUG, trnI-CAU, trnN-GUU, trnP-UGG, and trnW-CCA). The retention of functional genes provides insights into the role of intergenomic transfers in mitochondrial genome evolution and functional diversification.

### 3.5. Phylogenetic and Synteny Analysis

**Figure 5:**
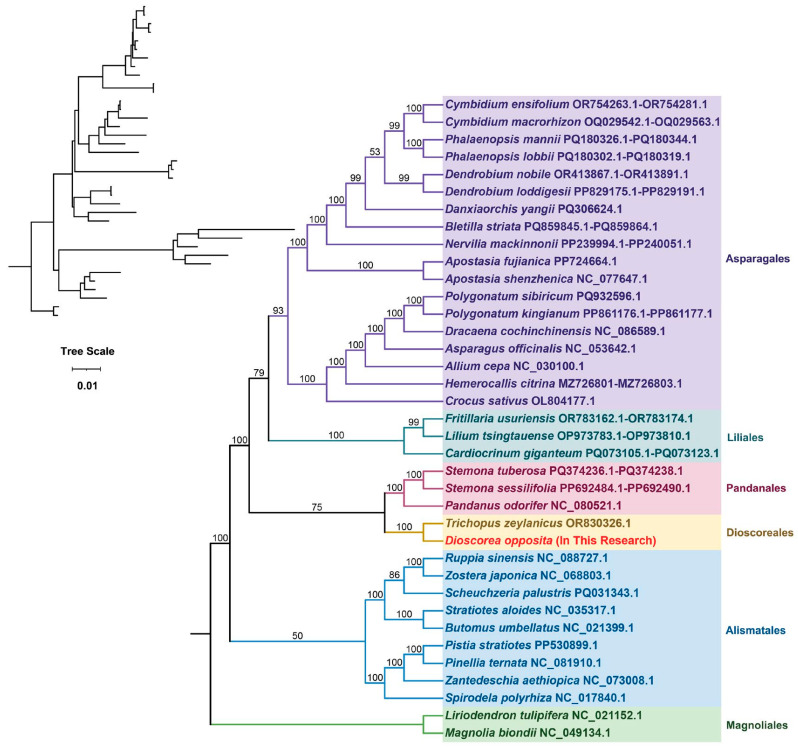
The phylogenetic analysis of the Tiegun yam with the 37 other represented land plants. Bootstrap support values are shown on each node, with colors representing the respective plant families.

Phylogenetic analysis in plants, which aims to elucidate evolutionary relationships among species, commonly employs mitochondrial DNA sequences, particularly those derived from conserved protein-coding genes within the mitochondrial genome, and is typically depicted as phylogenetic trees. In this investigation, a phylogenetic tree was constructed utilizing DNA sequences from 21 conserved mitochondrial PCGs sourced from 37 species across 6 angiosperm orders. The plant species under scrutiny are detailed in Table S16. The protein-coding genes scrutinized encompass *atp1*, *atp4*, *atp8*, *atp9*, *ccmB*, *ccmC*, *ccmFC*, *ccmFN*, *cob*, *cox1*, *cox2*, *matR*, *mttB*, *nad2*, *nad3*, *nad4*, *nad4L*, *nad5*, *nad6*, *nad7*, and *nad9*. Two mitochondrial genomes from the Magnoliales order were chosen as the outgroup. The resultant phylogenetic tree closely aligns with the prevailing Angiosperm Phylogeny Group (APG) classification system. Notably, the Tiegun yam was confidently positioned within the Dioscoreales order, forming a strongly supported clade with recognized members of this order, thereby reinforcing its taxonomic assignment and implying a shared evolutionary lineage with other Dioscoreales species (Figure 5).

**Figure 6:**
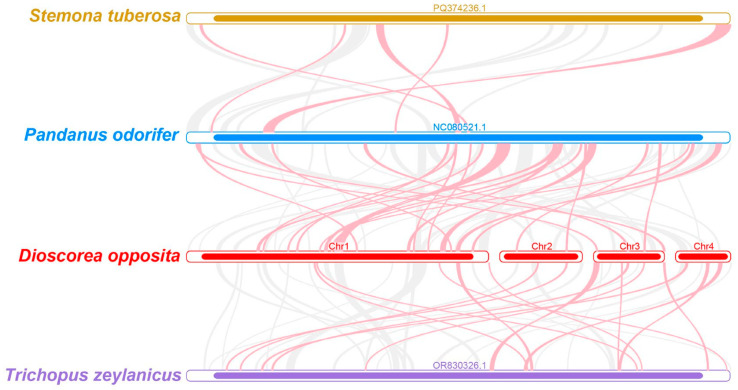
The Tiegun yam mitogenomes synteny. The bars represent the mitogenomes, while the ribbons illustrate the homologous sequences between neighboring species. Red areas highlight the locations of reversals, while gray areas indicate regions of strong homology. Blocks shared by species that are less than 0.5 kb in length are excluded, and regions lacking a common block are unique to specific species.

To elucidate the evolutionary history and structural dynamics of the Tiegun yam mitogenome, a collinearity analysis was conducted by comparing it with the mitochondrial genomes of three closely related species (Figure 6, Table S17). In the resulting plot, red arcs denote genomic inversions, while gray arcs represent high-confidence homologous regions. Collinear blocks shorter than 0.5 kb were excluded from visualization for clarity. The analysis identified multiple homologous collinear blocks present across all four species; however, their order and orientation varied significantly. Specifically, the Tiegun yam mitogenome displayed a high frequency of inversions and translocations, with seven major rearrangements involving segments longer than 5 kb. These structural variations indicate substantial reorganization of the mitochondrial genome during evolution. The observed lack of large-scale synteny conservation, even among closely related species, underscores the dynamic nature of plant mitochondrial genomes and their rapid evolution in terms of genome architecture.

### 3.6. RNA Editing Sites in PCGs

**Figure 7:**
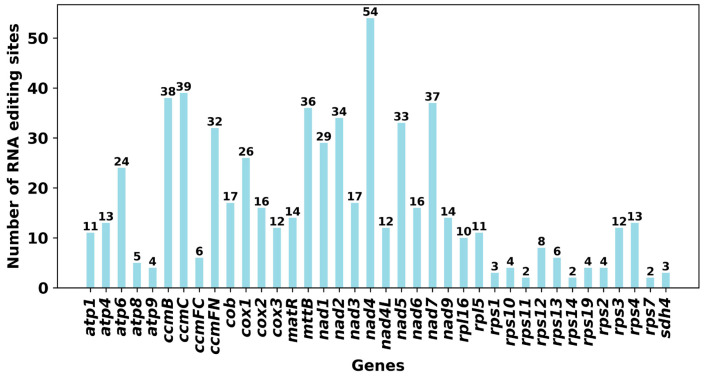
Number of RNA editing sites identified in each PCG of the Tiegun yam mitochondrial genome.

RNA editing is a prevalent post-transcriptional modification process observed in the mitochondria of higher plants, playing a crucial role in organellar gene expression. This process entails the targeted deamination of cytidine (C) to uridine (U) in RNA transcripts, resulting in alterations to the nucleotide sequence compared to the genomic template. Notably, these C-to-U editing events primarily occur at the second codon position and are typically precise. By restoring evolutionarily conserved amino acid residues, RNA editing contributes to enhancing the conservation of mitochondrial protein sequences across species, thereby increasing the sequence identity of orthologous proteins across different taxonomic groups. Previous research on plant mitochondria indicates that around 92% of RNA editing sites lead to modifications in the predicted protein sequence [47]. The most prevalent alteration involves the replacement of hydrophilic amino acids with hydrophobic ones, which is believed to support protein structural stability and correct folding [48]. Moreover, RNA editing has the capacity to introduce start and stop codons that are not originally present in the genomic DNA, thereby facilitating the synthesis of functional and evolutionarily conserved proteins [49]. This study focused on identifying RNA editing occurrences in 39 distinct PCGs within the mitochondria of the *D. opposita* (Table S15). By applying a cutoff threshold of 0.9, we detected a collective total of 723 potential RNA editing sites within these mitochondrial PCGs, all characterized by C-to-U substitutions. Notably, the gene *nad4* exhibited the highest frequency of editing sites at 60, with *ccmB* and *mttB* following closely behind at 46 sites (Figure 7).

## 4. Discussion

The plant mitochondrial genome is recognized for its intricate structure, substantial size variability, and frequent intergenic recombination. This study introduces the initial comprehensive assembly and comparative analysis of the mitochondrial genome of the Tiegun yam, a species of considerable medicinal and nutritional value. Our results not only clarify the distinctive architectural characteristics of its mitochondrial genome but also offer novel evolutionary perspectives on the phylogenetic status of this species within Dioscoreales and angiosperms in general.

### 4.1. Multichromosome Architecture: A “Fragmentation” Strategy of the Tiegun Yam Mitochondrial Genome

The mitochondrial genome of Tiegun yam is organized into four circular chromosomes (Figure S1), a configuration that is uncommon among angiosperms and exemplifies an intriguing evolutionary approach to genomic complexity. This multi-chromosomal arrangement, presented here for the first time in the monocot order Dioscoreales, contrasts with the prevalent single-circular structure observed in model organisms such as *A*. *thaliana* and *O*. *sativa* [20,49].

Homologous recombination, predominantly instigated by repetitive sequences, significantly influences the structural configuration of plant mitochondrial genomes [50]. Our investigation validates this concept by identifying a profusion of SSRs and lengthy dispersed repeats throughout all four chromosomes (Figure 3). Notably, a sizable ~24.8 kb direct repeat located on Chr1 emerges as a probable hotspot for intragenomic recombination. In numerous plant species, such extensive repeats promote frequent recombination occurrences, fostering the generation of subgenomic circular molecules and enhancing structural variability [51]. We posit that the stable, self-sufficient nature of the four circular chromosomes in Tiegun yam results from a deliberate “fragmentation” approach. This assertion is underpinned by the consistent sequencing coverage and the presence of intact origin of replication-associated sequences on each chromosome, conclusively refuting any assembly anomalies.

This fragmented architecture may provide a selective advantage, especially concerning the species’ long-term asexual reproduction, such as through bulbils. By physically segregating extensive repetitive regions onto distinct, autonomously replicating molecules, this strategy could alleviate the recombination burden, thereby minimizing the risk of large-scale, deleterious genomic rearrangements that may occur from homologous recombination between repeats co-localized on a single chromosome [52]. This mechanism offers a credible explanation for the stable maintenance of a complex genome structure in Tiegun yam.

### 4.2. Extremely Conserved Coding Genes and a Highly Specialized Codon Usage Bias

Despite its intricate structure, the mitochondrial genome of Tiegun yam preserves the core set of 39 conserved protein-coding genes characteristic of angiosperms, highlighting the robust purifying selection acting on essential respiratory functions. Notably, we observed a significant bias in codon usage for specific amino acids, exemplified by a preference for GCU in encoding alanine (RSCU = 1.64) (Figure 3).

Codon usage bias may result from mutational pressure or natural selection favoring translational efficiency and accuracy. It is crucial to emphasize that these conclusions are derived exclusively from in silico analyses. The functional implications of this observed bias remain uncertain. Future transcriptomic and proteomic studies are necessary to determine whether this codon usage pattern correlates with increased gene expression or affects the organism’s fitness, as has been explored in other plant systems such as Zea mays (maize) [53].

### 4.3. Chloroplast-Derived Sequence Migration: A New Case of Mitochondrial-Chloroplast “Cross-Compartment DNA Transfer”

Our study identified 18 mitochondrial plastid DNA (MTPT) fragments, collectively measuring 10.1 kb, which constitutes 2.04% of the mitochondrial genome (Figure 4). This proportion falls within the median range reported for various plant species. Importantly, eight apparently intact chloroplast-derived protein-coding genes were detected within these MTPTs.

Intact genes indicate potential functional gene transfer, a phenomenon observed in plant lineages. However, the presence of a sequence alone does not verify its functionality. The expression and functionality of these acquired genes within the mitochondrial environment are yet to be determined, posing a crucial area for future investigation. These MTPTs offer compelling evidence of continuous intercompartmental DNA movement, underscoring the ever-changing landscape of plant organellar genomes. Exploring these transfers sheds light on potential evolutionary adaptations, wherein chloroplast sequences could be repurposed for novel functions within the mitochondrion.

### 4.4. Phylogenetic Position and RNA Editing Characteristics: Deepening the Understanding of Dioscoreales Evolution

Phylogenetic analysis of 21 conserved mitochondrial protein-coding genes generated a robust evolutionary tree encompassing 37 species (Figure 5). The findings unequivocally positioned *D*. *opposita* within the Dioscoreales order, aligning with APG taxonomy and thereby strongly affirming its taxonomic classification. Nonetheless, collinearity analysis (Figure 6) unveiled substantial genomic rearrangements and structural disparities between the mitochondrial genome of the Tiegun yam and those of its close relatives (e.g., Stemona tuberosa, Pandanus odorifer, Trichopus zaylanicus), characterized by markedly different arrangements of collinear blocks. It is imperative to exercise caution in interpreting these outcomes due to the limited representation of mitochondrial genomes across Dioscoreales. Augmented taxonomic sampling is imperative to elucidate the structural evolutionary patterns and rates within this taxonomic group.

A total of 723 C-to-U RNA editing sites were predicted, a figure falling between the reported numbers in rice (~500) and grape (~800). Notably, *nad4* and *ccmC* were identified as hyper-edited genes, displaying a conserved distribution of editing hotspots akin to maize and tobacco. Previous studies have shown that *nad4* and *ccmC* play critical roles in mitochondrial function, particularly in cytochrome c biogenesis and respiratory chain activity. The *nad4* gene encodes a subunit of mitochondrial Complex I (NADH dehydrogenase), which is essential for electron transfer and ATP synthesis. RNA editing in nad4 could lead to amino acid alterations, influencing the subunit’s assembly, stability, or catalytic activity, thereby modulating respiratory efficiency. Similarly, *ccmC* is involved in the cytochrome c maturation (Ccm) system, specifically in the heme lyase pathway that facilitates the covalent attachment of heme to apocytochrome c. RNA editing may affect the structure or function of the CcmC protein, potentially disrupting heme handling or apocytochrome c binding, and ultimately impairing the production of mature cytochrome c. Since cytochrome c acts as an electron shuttle between Complex III and IV, any defect in its maturation could compromise mitochondrial electron transport and energy production. Similarly, ccmC is involved in the cytochrome c maturation (Ccm) system, specifically in the heme lyase pathway that facilitates the covalent attachment of heme to apocytochrome c. RNA editing in ccmC may impact the structure or function of the CcmC protein, possibly disrupting heme handling or apocytochrome c binding, which in turn could impair the biogenesis of functional cytochrome c. Since cytochrome c acts as an electron shuttle between Complex III and IV, any defect in its maturation could compromise mitochondrial electron transport and energy production. Remarkably, 8.7% of the editing sites were located at the first codon position, potentially leading to the creation of new start or stop codons. While these sites suggest an additional layer of post-transcriptional regulation, all predictions were computational. Thus, experimental validation, such as transcriptome sequencing, is essential to confirm the existence, frequency, and biological significance of RNA editing in this species. To advance these findings, future research should concentrate on two primary areas. First, transcriptomic validation is necessary to confirm the predicted RNA editing sites, evaluate the expression and potential functionality of MTPT-derived genes, and verify the transcriptional activity across all mitochondrial chromosomes. Second, investigating population-level diversity through the sequencing of mitochondrial genomes from various individuals and related species will be vital for understanding the structural stability, inheritance patterns, and evolutionary dynamics of this distinctive multi-chromosomal system.

## 5. Conclusions

This study presents the first complete mitochondrial genome of *D. opposita*, revealing a four-circular structure with a total length of 493,268 bp and a GC content of 45.67%. A total of 723 potential RNA editing sites were identified across 39 protein-coding genes (PCGs), all involving C-to-U substitutions. Strikingly, the *nad4* gene in Tiegun yam harbors 60 RNA editing sites the highest number reported in any crops to date-implying a central role in energy metabolism and environmental adaptation. These findings not only provide fundamental genomic resources but also offer practical applications for yam improvement. The unique structural features serve as reliable DNA markers for authenticating genuine Tiegun yam products, thereby combating market adulteration. Additionally, the extensive RNA editing sites, particularly in *nad4*, present valuable targets for developing molecular markers associated with stress adaptation, facilitating the selection of genotypes with optimized mitochondrial function for breeding programs. As the first multi-chromosomal mitochondrial genome in *Dioscoreales*, this assembly establishes a critical reference for evolutionary studies and functional genomics research. Collectively, these genomic insights lay a foundation for enhancing sustainable cultivation, genetic conservation, and commercial utilization of this important medicinal and food crop, potentially contributing to food security and agricultural sustainability in yam-producing regions.

## Figures and Tables

**Table 1:** Mitochondrial genome overview.

Contigs	Length (bp)	GC Content (%)
Chromosome 1–Chromosome 4	493,268	45.67
Chromosome 1	281,418	46.02
Chromosome 2	85,821	44.75
Chromosome 3	67,763	45.11
Chromosome 4	58,266	45.99

**Table 2:** Genes predicted in the mitogenoms of the Tiegun yam.

Group of Genes	Name of Genes
ATP synthase	*atp*1, *atp*4, *atp*6, *atp*8, *atp*9
NADH dehydrogenase	*nad*1, *nad*2, *nad*3, *nad*4, *nad*4L, *nad*5, *nad*6, *nad*7, *nad*9
Cytochrome b	*cob*
Cytochrome c biogenesis	*ccm*B, *ccm*C, *ccm*FC, *ccm*FN
Cytochrome c oxidase	*cox*1, *cox*2, *cox*3
Maturases	*mat*R
Protein transport subunit	*mtt*B
Ribosomal protein large subunit	*rpl*2, *rpl*5, *rpl*16
Ribosomal protein small subunit	*rps*1, *rps*2, *rps*3, *rps*4, *rps*7, *rps*10, *rps*11, *rps*12, *rps*13, *rps*14, *rps*19
Succinate dehydrogenase	*sdh*4
Ribosome RNA	*rrn*5 (×2), *rrn*18 (×2), *rrn*26 (×2)
Transfer RNA	*trn*A-UGC, *trn*C-GCA, *trn*D-GUC, *trn*E-UUC, *trn*F-GAA, *trnf*M-CAU (×4), *trn*H-GUG, *trn*I-CAU (×2), *trn*K-UUU, *trn*M-CAU, *trn*N-GUU (×2), *trn*P-UGG, *trn*Q-UUG, *trn*R-ACG, *trn*S-GCU, *trn*S-UGA, *trn*V-GAC, *trn*W-CCA, *trn*Y-GUA

## Data Availability

Bioproject: The complete mitogenome sequence reported in this study has been submitted to the National Center for Biotechnology Information (NCBI) under BioProject PRJNA1344416, BioSample SAMN52640154. Illumina reads are available under SRR35773046 and Oxford Nanopore long reads under SRR35773047. The lncRNA sequencing data reported in this study have been deposited in the NCBI Sequence Read Archive (SRA) under accession SRR36601826.

## Supplementary Materials

The following supporting information can be downloaded at: https://www.mdpi.com/article/10.3390/biology15020133/s1, Table S1. Relative synonymous codon use of codons by individual amino acids in the Tiegun yam mitochondrial genome. Table S2. SSRs in the mitochondrial genome of the Tiegun yam chr1. Table S3. SSRs in the mitochondrial genome of the Tiegun yam chr2. Table S4. SSRs in the mitochondrial genome of the Tiegun yam chr3. Table S5. SSRs in the mitochondrial genome of the Tiegun yam chr4. Table S6. Tandem repeat sequences in the mitochondrial genome of the Tiegun yam chr1. Table S7. Tandem repeat sequences in the mitochondrial genome of the Tiegun yam chr2. Table S8. Tandem repeat sequences in the mitochondrial genome of the Tiegun yam chr3. Table S9. Tandem repeat sequences in the mitochondrial genome of the Tiegun yam chr4. Table S10. Dispersed repeat sequences in the mitochondrial genome of the Tiegun yam chr1. Table S11. Dispersed repeat sequences in the mitochondrial genome of the Tiegun yam chr2. Table S12. Dispersed repeat sequences in the mitochondrial genome of the Tiegun yam chr3. Table S13. Dispersed repeat sequences in the mitochondrial genome of the Tiegun yam chr4. Table S14. The homologous DNA fragment in the Tiegun yam mitochondrial genome. Table S15. RNA-editing in the Tiegun yam mitochondrial genome. Table S16. The closed species with the Tiegun yam. Table S17. Colinear analysis of the Tiegun yam. Figure S1. The assembly graph of the Tiegun yam mitogenome.
