# Supplementary figures and images for: The Complete Mitochondrial Genome of the Dioscorea opposita Thunb. cv. Tiegun, a Traditional Medicinal and Edible Crop
Source: Biology (Basel). 2026 Jan 12;15(2):133. doi: 10.3390/biology15020133 (PMC12837428; doi:10.3390/biology15020133)

Figure S1. The assembly graph of the Tiegun yam mitogenome.

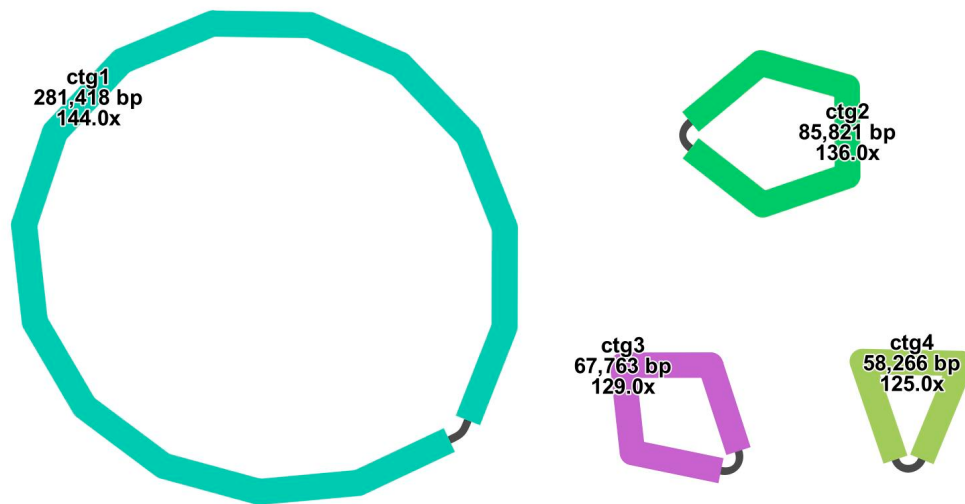

Supplement: Supplementary file 1 [file biology-15-00133-s001.zip › biology-3986964-Figure S1. The assembly graph of the Tiegun yam mitogenome.pdf]
